# 60. The sound of silence

**DOI:** 10.1093/rap/rky034.023

**Published:** 2018-09-20

**Authors:** Mrinalini Dey, Marwan Bukhari

**Affiliations:** Rheumatology, University Hospitals of Morecambe Bay NHS Foundation Trust, Lancaster, UNITED KINGDOM

**Introduction:** Cogan’s syndrome (CS) is a rare autoimmune vasculitis, involving the vestibulo-auditory and ophthalmic systems. It was first described by Morgan and Baumgarter in 1934, reporting non-syphilitic keratitis with vestibulo-auditory symptoms of hearing loss and dizziness. Ten years later, David Cogan, an ophthalmologist, described four patients with these symptoms, alongside vertigo, tinnitus, hearing loss and ocular disturbance. This combination of symptoms was later named Cogan’s syndrome. An atypical form of CS was later described, comprising other inflammatory eye lesions (including retinal artery occlusion and choroiditis). Atypical CS also includes vestibulo-auditory symptoms, occurring within two years of initial presentation and inconsistent with a Meniere’s-like phenomenon. Approximately 70% of such patients have vasculitis with systemic manifestations, usually relieved by corticosteroids. The condition is recognised as an autoimmune vasculitis, but there are no international diagnostic criteria, nor specific serological biomarkers; the separation of typical and atypical CS is commonly used. Autoantibodies against corneal, inner ear, and endothelial antigens have been identified, but there is limited data on their sensitivity and specificity for CS. The condition is not typically associated with ANCA, although it is classified as a variable vessel vasculitis in the revised 2012 International Chapel Hill Consensus Conference Nomenclature of Vasculitides. Only 250 cases of CS have been recorded, mainly affecting young Caucasian adults in the third decade, although it has also been reported in children and the elderly. One study on 78 patients had an age range of 5-63 years, with oldest age of onset being 47 years. Diagnosis can be difficult due to temporal separation of vestibulo-auditory and ocular symptoms. Delayed intervention with corticosteroids results in significant morbidity, including irreversible sudden hearing loss, and visual deterioration. Here we report a case of CS, comprising classical symptoms with atypical features. To our knowledge, this is the oldest recorded case of this condition.

**Case description:** A 75 year old lady presented to her GP with bilateral sudden deafness and dizziness with an unsteady gait, following a two-week history of early morning headaches with nausea but no vomiting. She was admitted under the ear, nose and throat (ENT) team, and an audiogram found sensorineural deafness, worse on the left than right. There were no signs of meningism, and tone, power, and reflexes were normal. She had positive nystagmus. The respiratory, cardiovascular and gastrointestinal systems were normal. During admission, she had several spikes in temperature, as well as worsening redness in her left eye. An ophthalmology referral was therefore completed. On examination, she was found to have left-sided nodular scleritis and bilateral hard fundal exudates, although pupils were equal and reactive. Intra-ocular pressures were normal. A fluorescein angiography showed colloid bodies over the posterior poles of both eyes. During admission, the lady had several investigations including full blood count, ESR, renal and liver function tests, bone marrow biopsy, abdominal ultrasound scan, MRI of the brain, lumbar puncture, and a full autoimmune screen. All were normal, aside from a raised ESR (108) and raised liver function tests. In light of the above test results, along with her scleritis and bilateral sudden onset sensorineural deafness, the ophthalmologists suspected Cogan’s syndrome and commenced her on high-dose steroids and referred for rheumatology review. Although her hearing did not return, she had marked improvement in her inflammatory markers and was discharged after one month on a reducing course of steroids, as well as steroid eye drops. She had regular follow-up with the rheumatology team. She continued to be profoundly deaf, and experienced intermittent dizziness, unsteadiness, and tinnitus, which worsened over the subsequent months leading to the requirement for a walking aid. ESR continued to decline and fundal exudates gradually cleared. Six months after her initial presentation, she had a further transient episode of scleritis in the left eye, after which she was reviewed by ophthalmology and advised to discontinue treatment. Due to worsening pains in several of her joints, an x-ray was performed of her hips, spine, and sacro-iliac joints with showed mild spondylolisthesis and facet joint change. An audiogram performed at this time showed no response for air or bone conduction, no different from the audiogram performed on initial admission. Despite learning to lip-read, she was struggling to cope with this aspect of her disease, and was therefore fitted with cochlear implants, which greatly improved her quality of life. She was off all steroid treatment just under two years after her first admission, with no renewed features of her disease including scleritis or repeated episodes of vasculitis. Three years following her initial episode, the patient was admitted from rheumatology clinic due to increasingly severe headaches over the preceding two months, with elevated inflammatory markers (ESR 99, CRP 127). The headaches were intermittent and localised to the left temporal region. An infection screen was negative and CT scan of the brain showed no new pathology. She was treated with a course of IV methylprednisolone, after which she showed clinical and biochemical improvement and was discharged. She continued to have intermittent flares of her Cogan’s syndrome, requiring further courses of steroids. She also had ongoing treatment and surgery for osteoporosis and bilateral cataracts respectively, likely induced by her high cumulative dose. Over the subsequent five years, the patient became increasingly frail and unsteady, experiencing muscle spasms leading to an inability to carry out activities of daily living, such as lifting herself out of the bath. Her steroids were reduced to zero, which improved the dizziness, but the frailty and declining mobility persisted. She began to have frequent falls, leading to several admissions, including for osteoporotic wedge fractures and femoral fracture, requiring a total hip replacement. In light of her worsening mobility, she was referred for review by the care of the elderly team. She was found to have a shuffling gait, mild bradykinesia, and increased tone, more so on the right, as well as a high systolic blood pressure of 180, with no postural drop. Over the subsequent months, she developed fine tremors. A diagnosis of vascular parkinsonism was made, and treatment with co-beneldopa commenced. In recent years, despite continued frailty, the patient has remained clinically well. She has developed some circulatory problems, including Raynaud’s phenomenon, as well as Sjogren’s syndrome, demonstrated by a very positive Schirmer’s test. Neither, however, requires treatment beyond conservative measures at present. As well as presenting as a typical case of Cogan’s syndrome, with atypical features, to our knowledge, she remains the oldest patient to be diagnosed with, and continuing to live with, this disease.

**Discussion:** Cogan’s syndrome requires a multi-disciplinary approach to management, and this is exemplified by this case. Our patient was diagnosed surprisingly quickly compared to others presenting with similar symptoms, with relatively efficient communication between ENT, ophthalmology, and rheumatology. There are few conditions that can cause both complete sensorineural hearing loss and visual deterioration, and they can of course be unrelated, but this case highlights the need to keep vasculitis and this particular condition in mind. This is especially important in this case given the age of the patient at presentation; the typical age of presentation, consistent with most autoimmune rheumatological conditions, is the third or fourth decade. Systemic vasculitis is a feature more consistent with the atypical form of this disease. Typical features in this case include the initial presentation of scleritis with bilateral sensorineural deafness. It is likely that the remitting-relapsing nature of this patient’s systemic vasculitis contributed to her subsequent vascular Parkinsonism, something that has not previously been described in the literature. It should not be assumed that neurological symptoms in these patients is solely due to their vasculitis, but it is important to consider when planning management, as well as closely monitoring inflammatory markers. Cogan’s syndrome has no formal diagnostic criteria or treatment guidelines, with management mostly based on anecdotal evidence. This leads to the question as to whether high-dose steroids are the most appropriate treatment for these patients in the long run. High-dose cumulative steroids leads to several complications, as demonstrated by this case, and it is worth considering whether alternative immunosuppressive agents would be more effective to prevent further episodes.

**Key Learning Points:** Cogan’s syndrome in its typical form comprises scleritis or keratitis, in association with sensorineural hearing loss. It is an important diagnosis to consider in patients presenting with these symptoms with little temporal separation. Atypical Cogan’s syndrome involves other inflammatory ocular conditions, as well as systemic vasculitis and more pronounced vestibular symptoms, although it is important to remember that these can occur in conjunction with the typical form of the disease. Although more common in the third decade of life, Cogan’s should be considered in patients of all ages who present with the above constellation of symptoms. Vasculitis associated with Cogan’s may lead to neurological sequelae later in life, and these should be considered in the presence of new non-specific symptoms, such as general unsteadiness, confusion, and other focal neurology. Imaging of the brain is essential to rule out other causes. High-dose steroids remains the mainstay of treatment for this disease, and remains the most effective way of inducing remission.

**Disclosure: M. Dey:** None. **M. Bukhari:** Honoraria; MB has received honoraria for speaking, and has attended advisory boards with Bristol-Myers Squib, UCB Celltech, Roche/Chugai/Pfizer, Abbvie, Merck, Mennarini, Sanofi-Aventis, Eli-Lilly, and Novartis.. Grants/research support; MB has been sponsored to attend national and international meetings by UCB Celltech, Roche/Chugai, Pfizer, Abbvie, Merck, Mennarini, and Eli-Lilly.

